# Hydrogel-Impregnated Self-Oxygenating Electrospun Scaffolds for Bone Tissue Engineering

**DOI:** 10.3390/bioengineering10070854

**Published:** 2023-07-19

**Authors:** Robin Augustine, Vasilios K. Nikolopoulos, Gulden Camci-Unal

**Affiliations:** 1 Department of Chemical Engineering, University of Massachusetts, Lowell, MA 01854, USA; robin@robinlab.in (R.A.); vasiliosn01@gmail.com (V.K.N.); 2 Department of Surgery, University of Massachusetts Medical School, Worcester, MA 01605, USA

**Keywords:** bone tissue engineering, scaffolds, oxygen-generating materials, PCL, GelMA, CaO_2_

## Abstract

Bone defects resulting from trauma, disease, or aging present significant challenges in the clinic. Although biomaterial scaffolds for bone-tissue engineering have shown promising results, challenges remain, including the need for adequate mechanical strength and suitable bioactive agents within scaffolds to promote bone formation. Oxygen is a critical factor for successful bone formation, and low oxygen tension inhibits it. In this study, we developed gelatin methacryloyl (GelMA) hydrogel-impregnated electrospun polycaprolactone (PCL) scaffolds that can release oxygen over 3 weeks. We investigated the potential of composite scaffolds for cell survival in bone-tissue engineering. Our results showed that the addition of an increased amount of CaO_2_ nanoparticles to the PCL scaffolds significantly increased oxygen generation, which was modulated by GelMA impregnation. Moreover, the resulting scaffolds showed improved cytocompatibility, pre-osteoblast adhesion, and proliferation under hypoxic conditions. This finding is particularly relevant since hypoxia is a prevalent feature in various bone diseases. In addition to providing oxygen, CaO_2_ nanoparticles also act as reinforcing agents improving the mechanical property of the scaffolds, while the incorporation of GelMA enhances cell adhesion and proliferation properties. Overall, our newly developed self-oxygenating composite biomaterials are promising scaffolds for bone-tissue engineering applications.

## 1. Introduction

Bone defects can occur for various reasons, such as trauma, disease, or aging. Repairing bone defects using biomaterial scaffolds is a promising approach that has gained much attention in recent years. Electrospinning is a widely used technique to fabricate scaffolds with a high surface area-to-volume ratio and interconnected porous structure which can resemble the native extracellular matrix (ECM) of bone [1,2]. However, there are challenges to be addressed, such as the lack of adequate mechanical strength and the need for suitable bioactive agents to promote bone formation.

The importance of oxygen in bone formation has been widely recognized [3]. Oxygen is required for the metabolism of cells in the regenerating tissue, and low oxygen tension has been shown to inhibit osteogenic differentiation and bone formation [4]. Therefore, providing an adequate oxygen supply to the regenerating tissue is crucial for repairing bone defects [5]. This is particularly important in large bone defects, where the distance between the cells in the center of the defect and the surrounding tissue can be greater than the diffusion limit of oxygen [6]. In recent years, the use of oxygen-generating agents in tissue engineering has emerged as a potential solution [7]. In particular, calcium peroxide (CaO_2_) nanoparticles (CPN) have shown promise in enhancing bone regeneration due to their oxygen generating capacity [8,9,10]. CaO_2_ can release oxygen slowly in the presence of moisture. The reaction between CaO_2_ and water produces hydrogen peroxide (H_2_O_2_), which decomposes to release oxygen [11]. Oxygen supplementation to seeded cells from the CaO_2_ nanoparticles in the scaffold occurs through the reaction pathway highlighted below [5,12,13].

$${CaO}_{2}+2H_{2}O\overset{}{\to }Ca{\left(OH\right)}_{2}+H_{2}O_{2}$$

$${2H}_{2}O_{2}\overset{Catalase}{\to }{2H}_{2}O+O_{2}$$

This property makes CaO_2_ a promising material for use in bone-tissue engineering, as oxygen is essential for bone formation.

Electrospinning is a process that uses electricity to create ultrafine fibers from a liquid solution or melt. A high voltage is applied to the liquid, causing it to form a fine jet that stretches and solidifies into fibers. These fibers have various applications in fields such as materials science and biomedical engineering [14,15,16]. Electrospun polycaprolactone (PCL) scaffolds have been used in bone-tissue engineering due to their ability to mimic the ECM of bone, thereby providing a suitable environment for cells to adhere, proliferate, and differentiate [2,17,18]. PCL is a biocompatible and biodegradable polymer that can be processed into various configurations, such as electrospun nanofibers, which can enhance cell attachment and nutrient diffusion. However, there are also several drawbacks associated with the use of electrospun PCL scaffolds in bone-tissue engineering. Firstly, the mechanical strength of PCL scaffolds may not be comparable with that of natural bone [19]. Secondly, despite the structural similarity, PCL scaffolds may not fully mimic the functional attributes of natural bone ECM which can impact cell adhesion and proliferation. Thirdly, vascularization through the closely packed electrospun PCL scaffolds can be a slow process which affects oxygenation and cell proliferation. In this context, the use of CPN as an oxygen-generating material in electrospun scaffolds has been investigated to enhance bone-tissue regeneration [19,20]. A recent study reported on the fabrication of electrospun PCL scaffolds containing CaO_2_ for bone regeneration [21]. However, to improve the cell adhesion and proliferation on PCL scaffolds, which lack surface functional groups, the incorporation of a natural ECM-based material will be advantageous. Gelatin methacryloyl (GelMA) is produced by modifying gelatin through the methacrylation process, which involves the use of methacrylate anhydride [22,23]. This modification renders the polymer photocrosslinkable using photoinitiators such as Irgacure and UV light [22,24]. The GelMA impregnation into the PCL–CPN scaffolds helps improve cell adhesion and proliferation. 

The CPN in electrospun scaffolds have several potential benefits for bone-tissue engineering. Firstly, the slow release of oxygen from CPN can improve the oxygen supply to the cells in the scaffold and the surrounding tissue [25]. Oxygen has been shown to play a crucial role in bone regeneration by promoting the proliferation of bone related cells [7]. In addition, CPNs act as reinforcing agents to improve the mechanical properties of the scaffolds, which is important for bone-tissue engineering [11]. 

Thus, we developed electrospun PCL scaffolds loaded with CPN to address the challenges with inadequate oxygen supply in tissue engineering. The incorporation of CPN improved oxygen supply and enhanced mechanical properties. Additionally, the infiltration of GelMA supported cell adhesion and proliferation. Our self-oxygenating scaffolds hold promise for bone-tissue engineering applications requiring osteogenic cell proliferation under hypoxia and improved mechanical strength.

## 2. Materials and Methods

### 2.1. Materials

We obtained calcium chloride (CaCl_2_), sodium hydroxide (NaOH), H_2_O_2_, 30%, ammonium hydroxide solution (50%), gelatin, methacrylate anhydride, and PCL with 80,000 molecular weight from Sigma-Aldrich. Dichloromethane (DCM) was from Merck Millipore. A live/dead cell imaging kit (Invitrogen-Molecular probes) and Alizarin red S was obtained from Thermo Fisher Scientific. Polyethylene glycol 200 (PEG200) was from Fisher Scientific. The following were sourced from Gibco: phosphate-buffered saline (PBS), Dulbecco′s modified Eagle’s medium (DMEM), catalase (H_2_O_2_:H_2_O_2_ oxidoreductase, EC 1.11.1.6), penicillin streptomycin solution, trypsin-ethylenediaminetetraacetic acid (EDTA), and fetal bovine serum. The tissue culture flasks and plates used in this research were from GenClone, Genesee Scientific (San Diego, CA, USA). 

### 2.2. Synthesis of CPN

**Figure 1:**
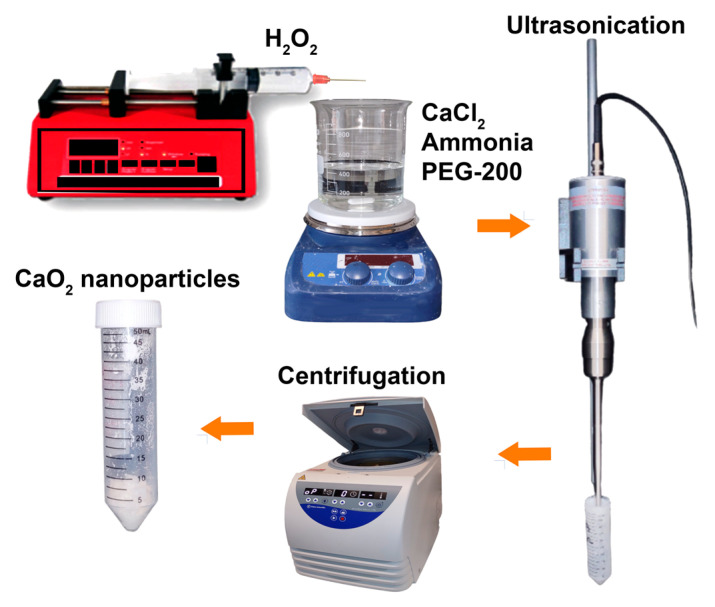
Scheme showing the process of CPN synthesis. The process includes the gradual addition of H_2_O_2_ to a mixture of CaCl_2_/Ammonia/PEG-200 while stirring, followed by ultrasonication, centrifugation, washing, and drying.

CPN were synthesized via a precipitation method with minor modifications [26,27,28,29]. The experiment involved the synthesis of nanoparticles using CaCl_2_, ammonium hydroxide solution, polyethylene glycol (PEG-200), and H_2_O_2_. For this, 6 g of CaCl_2_ was dissolved in 30 mL double-distilled water followed by the addition of 20 mL ammonia solution and 240 mL of PEG 200 (Figure 1). The mixture was then stirred constantly while adding 70 mL of 30% H_2_O_2_ at a rate of 0.25 mL per minute. The solution was stirred for 3 h and turned yellowish in color. NaOH solution was added to adjust the pH to 11.5, and the resulting nanoparticle suspension was yellow-white in color. The nanoparticles were separated by centrifugation, washed quickly with dilute NaOH solution, deionized water, and ethanol and then dried at 80 °C for 24 h in a hot air oven.

Scanning electron microscopic (SEM) analysis of synthesized nanoparticles was performed to assess their morphological features. Prior to SEM analysis, the samples were coated with platinum using a Leica SCD500 sputter coater. SEM analysis was performed at 3 kV. 

To assess the crystalline structure and crystallinity of the synthesized nanoparticles, X-ray diffraction (XRD) analysis was performed. XRD data were collected over a 2θ range of 5–80° using a PANalytical X’Pert Pro equipped with CuKα radiation (Malvern Panalytical, Malvern, UK) that had an energy of 8.04 keV and a wavelength of 1.54 Å. The instrument was operated at a voltage of 40 kV and a current of 25 mA.

The Bruker Alpha II ATR spectrometer (Billerica, MA, USA) was used to obtain the FT-IR spectra of the nanoparticles. The spectra were collected in the range of 450–4000 cm^−1^.

### 2.3. Fabrication of Electrospun GelMA-PCL–CPN Scaffolds

**Figure 2:**
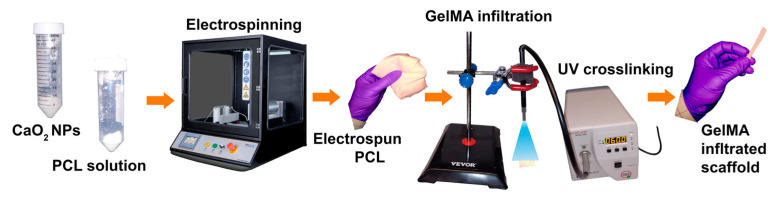
Fabrication process of GelMA-PCL–CPN scaffolds. The electrospun PCL–CPN scaffolds were fabricated by electrospinning. Fabricated PCL–CPN scaffolds were impregnated with GelMA and crosslinked using a UV source.

Figure 2 presents a scheme for scaffold fabrication through the electrospinning of a CPN-containing PCL solution, subsequent infiltration of GelMA, and crosslinking. The first step in the scaffold fabrication process is the electrospinning of a CPN-containing PCL solution. The electrospinning process involves the use of a high-voltage power supply to create an electric field that can generate a polymer jet from the PCL solution [30]. The next step in the scaffold fabrication process is the infiltration of GelMA into the electrospun scaffold. GelMA is a chemically modified biopolymer that can improve the biocompatibility, mechanical properties, and cell adhesion of the scaffold [31,32]. The incorporation of GelMA into the electrospun scaffold was achieved by immersing the scaffold in a GelMA solution and allowing it to absorb the solution. The final step in the scaffold fabrication process is the crosslinking of the GelMA. Crosslinking was achieved by exposing final construct to UV light, which forms covalent bonds between the GelMA molecules. Crosslinking can improve the mechanical properties and stability of the scaffold, as well as preventing the GelMA from leaching out of the scaffold [33]. The following section presents a detailed description of the experiments.

Electrospinning: PCL polymer (Sigma-Aldrich, St Louis, MO, USA) was dissolved in dichloromethane (Sigma-Aldrich, USA) to obtain a 12% (*w*/*v*) solution. The solution was stirred overnight to ensure complete dissolution. One to 6% CPN were dispersed in PCL solution. The electrospinning process was carried out using a Inovenso electrospinning machine (model: NS1 PLUS, Turkey) providing high voltage power supply at a voltage of 18 kV, a flow rate of 1 mL/h, and a distance of 15 cm between the needle and the collector. 

Plasma treatment of PCL scaffolds: To improve the surface wettability and enhance the adhesion of GelMA, the PCL scaffolds were treated with plasma before infiltration with GelMA. The plasma treatment was performed using a plasma cleaner (Harrick Plasma, model no. PDC-001-HP, USA), which generated a low-pressure air plasma. The PCL scaffolds were placed in the plasma cleaner and exposed to the plasma for a 10 s duration of time under a constant flow rate of ambient air. The plasma-treated PCL scaffolds were then immediately used for GelMA infiltration.

GelMA impregnation: GelMA was synthesized according to the protocol described by Bulcke et al. (2000) [23] and used in our published works [34,35]. Briefly, gelatin (Type A, Sigma-Aldrich, USA) was dissolved in PBS (pH 7.4) at a concentration of 10% (*w*/*v*), and the solution was heated to 60 °C with constant stirring. Methacrylic anhydride (Sigma-Aldrich, USA) was added dropwise to the solution, and the reaction was allowed to proceed for 3 h at 50 °C. The solution was then dialyzed against deionized water for 7 days and lyophilized. Photoinitiator solution was prepared by adding Irgacure 2959 (2-hydroxy-4′-(2-hydroxyethoxy)-2-methylpropiophenone) to PBS at a 0.5 weight percent by volume concentration, and heating at 70 °C for at least 30 min until dissolved. GelMA solution was then prepared by dissolving lyophilized GelMA in photoinitiator at a concentration of 5% (*w*/*v*) and heating to 70 °C until the GelMA was fully dissolved in the photoinitiator solution. The plasma-treated PCL scaffolds were vortexed in the GelMA solution for 2 min under sterile conditions using an analog vortex (Fisher Brand, model no. 9454FIALUS, USA). The excess GelMA solution was gently removed, and the 1 cm × 1 cm GelMA-infiltrated PCL scaffolds were crosslinked with ultraviolet (UV) light (OmniCure Series 2000, model no. S2000-XLA-9566, Canada) for 1 min on each side. The scaffolds used to test their strength were rectangular, measuring 1 × 6 cm. To make GelMA stable, they underwent a crosslinking process for a total of six minutes. During this process, each side of the scaffold was treated separately for three minutes each. Since the samples were long, for getting required UV exposure, both edges were treated for one minute, and the middle of the scaffold was treated for one minute as well.

The crosslinked scaffolds were washed with sterile PBS and stored at 4 °C until further use. Prepared scaffolds were labelled as GelMA–PCL (GelMA impregnated blank PCL scaffolds), GelMA-PCL–CPN-1 (GelMA impregnated PCL scaffolds containing 1% *w*/*w* CPN), GelMA-PCL–CPN-2 (GelMA impregnated PCL scaffolds containing 2% *w*/*w* CPN), GelMA-PCL–CPN-3 (GelMA impregnated PCL scaffolds containing 3% *w*/*w* CPN), GelMA-PCL–CPN-4 (GelMA impregnated PCL scaffolds containing 4% *w*/*w* CPN), GelMA-PCL–CPN-6 (GelMA impregnated PCL scaffolds containing 6% *w*/*w* CPN).

### 2.4. Characterization of the Scaffolds

The morphology of the scaffold was observed by SEM (JEOL JSM 7401F, Japan). SEM analysis was performed on GelMA–PCL and GelMA–PCL–CPN scaffolds to assess their morphological features. The scaffolds were about 0.5 mm thick, 5 cm long, and 2 cm wide. Each scaffold was sectioned using scissors and mounted on an SEM stub. Prior to SEM analysis, the samples were coated with platinum using a Leica SCD500 sputter coater. SEM analysis was performed at 3 kV. The diameter of the fibers was determined using the NIH ImageJ software, which measured 100 random positions on each sample and calculated the average diameter based on these measurements. The dry weight of scaffolds was measured using a weighing balance before and after GelMA impregnation to quantify the weight gain. Furthermore, FTIR analysis of the scaffolds was performed using a Bruker Alpha II ATR spectrometer (Billerica, MA, USA). The spectra were collected in the range of 450–4000 cm^−1^.

The presence of calcium in the PCL–CPN scaffolds was detected by Alizarin red staining. The scaffolds were incubated with 2% Alizarin red solution (pH 4.2) for 3 h at room temperature with gentle shaking. After incubation, the scaffolds were washed with deionized water to remove excess dye and air-dried. The presence of calcium in the scaffolds was visualized as red staining under a Stemi 508 stereo microscope (Carl Zeiss AG, Oberkochen, Germany) attached with an Axiocam 208 color camera (Carl Zeiss AG, Oberkochen, Germany). Each scaffold in the well was treated with a 10% (*w*/*v*) NaOH solution to dissolve the calcium deposits, and then the plate was incubated at room temperature for 3 h with gentle shaking. The resulting solution was used to measure the amount of Alizarin red that had bound to the calcium in the scaffolds. This involved adjusting the pH of the solubilized solution to 10.0 with a 10% (*w*/*v*) ammonium hydroxide solution, followed by heating at 85°C for 10 min. After cooling to room temperature, the absorbance was measured at 405 nm using a SpectraMax M3, (Molecular Devices, San Jose, CA, USA) microplate reader, and the resulting absorbance values for each sample were measured.

### 2.5. Tensile Testing

The mechanical properties of the PCL–CPN scaffolds were evaluated using a Universal Testing Machine (Shimadzu-EZ-LX, Kyoto, Japan) in accordance with the ASTM D 882 standard. The scaffolds were cut into rectangular strips (approximately 6 cm × 1 cm × 0.5 mm) using scissors. The samples were allowed to swell in PBS for 1 hour before the testing. The samples were mounted onto the grips of the testing machine and pulled at a constant rate of 2 mm/min until failure. A 500-N load cell was used for measuring the force. The stress and strain were recorded, and the tensile strength, elongation at break, and Young’s modulus were calculated using the recorded data. At least five samples were tested for each group, and the average values were calculated.

### 2.6. Swelling Study

The swelling ratio of the 1 × 1 cm GelMA impregnated PCL scaffolds of the evaluated CPN concentrations (0%, 1%, 2%, 3%, 4%, 6%) was analyzed according to the following procedure. First, the dry weight of all scaffolds was measured and recorded. The scaffolds were then submerged in 1 mL PBS at room temperature and were allowed to absorb the solution. The scaffolds were then removed from PBS at various intervals post-soak. Wet weights were again measured and recorded. The swelling ratio for the various measures was calculated according to the following formula:

$$SR=\left(\frac{Soak\;Wt.-Dry\;Wt.}{Dry\;Wt.}\right)\;*\;100$$

where *SR* is the swelling ratio of a scaffold as a percent, *Soak Wt.* is the post soak weight of the scaffold in grams, and *Dry Wt.* is the initial dry weight of the scaffold in grams.

### 2.7. Testing Oxygen Release from the Scaffolds under Hypoxic Conditions

To investigate the kinetics of oxygen release from GelMA-impregnated CPN-loaded scaffolds, oxygen-release experiments were conducted in hypoxic conditions with and without cells. To measure the percentage of dissolved oxygen in the media in which either cell-free or cell-seeded scaffolds are placed under hypoxia, 25 mg samples were placed in 24-well plates containing 1 mL of DMEM media. The dissolved oxygen was measured every 24 h for a period of 21 days. The oxygen level was maintained at 4–5% to study the release kinetics independent of atmospheric oxygen levels. To achieve this, a hypoxia chamber from STEMCELL Technologies in Vancouver, Canada was used in accordance with the manufacturer’s instructions for setting up the hypoxia. Catalase (1 mg/mL), an enzyme that increases the conversion efficiency of H_2_O_2_ to water and oxygen was used in the media. The oxygen levels were measured using a an optical oxygen sensing probe (NeoFox, Ocean Optics, FL, USA).

### 2.8. Testing of Cell Viability and Cell Proliferation on the Scaffold under Hypoxic Conditions

Cell culture: Pre-osteoblast cell lines, MC3T3-E1, were obtained from ATTCC and cultured in Dulbecco’s modified Eagle’s medium (DMEM) supplemented with 10% fetal bovine serum (FBS) and 1% penicillin/streptomycin. The cell culture experiments were performed using a 24-well plate with scaffold placed in the center of each well. The pre-osteoblast-seeded scaffolds (100,000 cells per cm^2^) were incubated under hypoxic conditions (ref. Section 2.7 for details) at 37 °C and 5% CO_2_ for a certain period, typically 3 weeks, with medium changes every 2–3 days. Cell viability and proliferation were assessed using MTT assay and live/dead assay. The cell-seeded scaffolds were compared to controls, including scaffolds without CPN to assess the effect of the scaffold on cell behavior and potential for bone regeneration.

## 3. Results and Discussion

The results of this study demonstrate that the use of electrospun PCL–CPN scaffolds impregnated with GelMA is a potentially suitable biomaterial for bone-tissue engineering applications. The sustained release of oxygen from the CPN improved cell viability and proliferation, while the improved mechanical properties and swelling of the scaffolds suggest that the PCL–CPN scaffolds have the potential to be used in tissue engineering.

### 3.1. Characterization of the CPN

The CPN was synthesized successfully using the precipitation method. The morphology of the nanoparticles was determined using scanning electron microscopy (SEM), the crystalline structure was determined using XRD, and the functional groups were determined using Fourier-transform infrared spectroscopy (FTIR).

**Figure 3:**
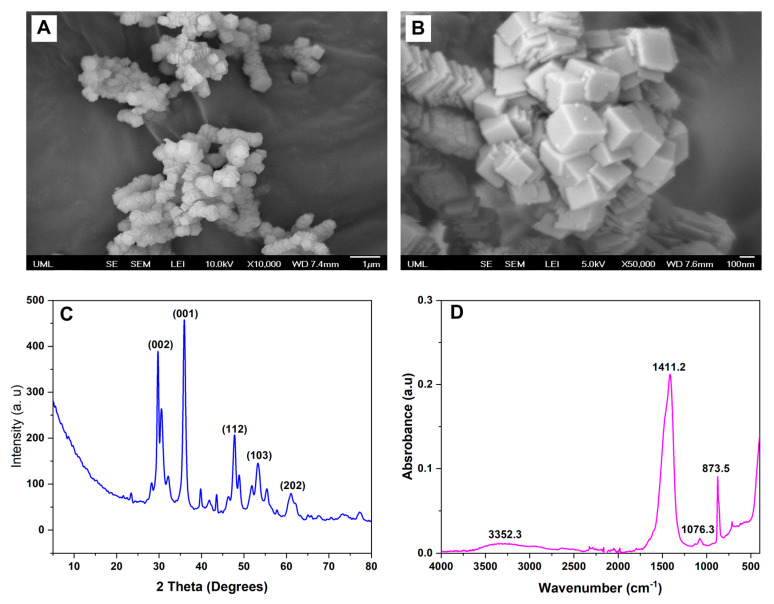
The characterization of synthesized calcium peroxide (CaO_2_) nanoparticles (CPN). Scanning electron microscopy image of CPN morphology at (**A**) low magnification and (**B**) at high magnification. (**C**) X-ray diffraction pattern of synthesized CPN, displaying the characteristic diffraction patterns of CaO_2_. (**D**) Fourier-transform infrared spectrum of CPN, exhibiting the characteristic peaks of CaO_2_.

SEM images showed that the CPN had a cubical morphology with a size range from 74 to 114 nm (Figure 3A,B). The nanoparticles were found to be well dispersed and agglomerate-free. The nanoparticles had a relatively uniform size distribution, with no evidence of any significant size variation within the sample.

The XRD pattern of the nanoparticles exhibited several distinct peaks, indicating that the particles were crystalline in nature (Figure 3C). The diffraction peaks at 2θ values of 16.9°, 24.3°, 32.6°, 34.5°, 45.3°, and 50.3° corresponded to the (110), (200), (211), (220), (310), and (222) planes of the calcium peroxide nanoparticles, respectively. For instance, a study by Madan et al. investigated the XRD pattern of calcium peroxide nanoparticles synthesized using a chemical precipitation technique [26]. The XRD pattern exhibited similar peaks and corresponding planes of the calcium peroxide nanoparticles as in previously conducted studies, which were adopted here for the synthesis of CPN [26,27,28]. Similarly, other studies which investigated the XRD pattern of calcium peroxide nanoparticles synthesized via a co-precipitation method also exhibited distinct peaks at similar 2θ values, indicating the cubic crystal structure of the nanoparticles [36,37]. These results indicate that the nanoparticles synthesized in this study possessed a crystalline nature and exhibited a cubic crystal structure. The narrow width and high intensity of the diffraction peaks suggest a relatively large crystallite size and high degree of crystallinity in the particles. The absence of non-indexed patterns further confirms the nanoparticles’ high purity.

FTIR spectra revealed characteristic peaks of the CPNs were consistent with previous studies (Figure 3D) [38,39]. The broad band observed at around 3400–3200 cm^−1^ corresponded to the stretching vibration of the –OH group, which may be attributed to the adsorbed water molecules and the hydroxyl groups on the CPN [38]. Moreover, an absorption band was observed at around 3385 cm^−1^, which is attributed to the ether group of PEG-200 bound on the CPN. The peak observed at 872 cm^−1^ indicated the presence of the peroxide group (-O-O-) in CPNs [40]. The absorption band observed at around 1075 cm^−1^ was attributed to the ether group (-C-O-C-) of PEG-200 bound on the CPNs, which was also consistent with previous studies [41]. Additionally, peaks observed at around 1425 cm^−1^ and 500 cm^−1^ were assigned to the O-Ca-O vibrations, which are characteristic of CPNs [39,42].

### 3.2. Morphology and Fiber Diameter Distribution of the Self-Oxygenating GelMA-Impregnated PCL Scaffolds 

**Figure 4:**
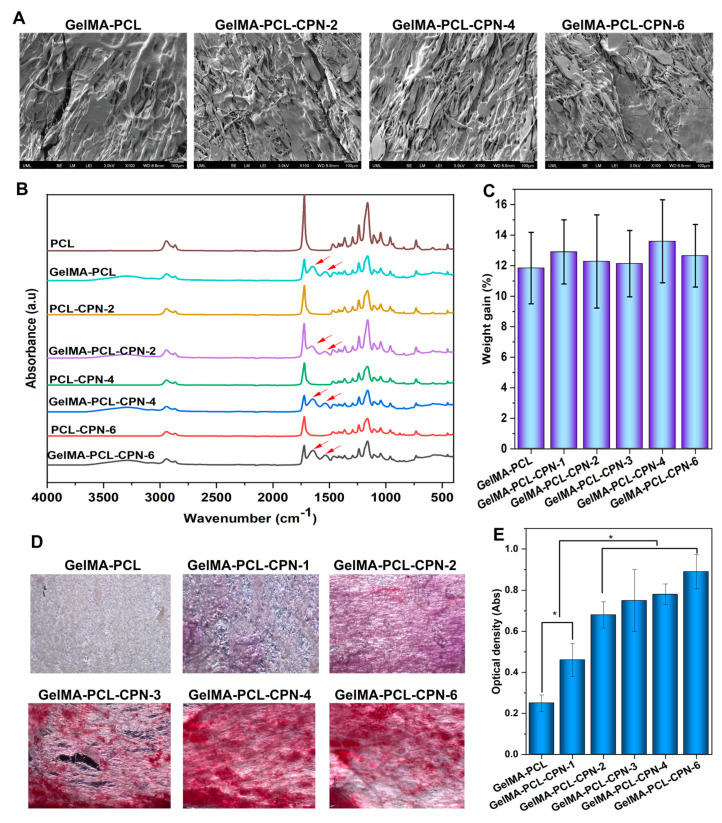
The characterization of fabricated GelMA–PCL–CPN scaffolds. (**A**) A scanning electron microscopy image is shown to depict the scaffold morphology. (**B**) The FTIR spectrum of the scaffolds is presented, exhibiting the characteristic peaks of GelMA. (**C**) The weight gain of the scaffold after GelMA infiltration is shown, indicating successful penetration of GelMA inside the PCL–CPN scaffolds. (**D**) Alizarin red staining shows a concentration-dependent increase in calcium ions in GelMA–PCL–CPN scaffolds, indicating the successful loading of the CPN in the scaffolds. (**E**) Quantification of the Alizarin Red S-calcium complex is shown by absorbance measurements at 405 nm. Statistical significances are indicated by * *p* < 0.05.

The morphology of the scaffolds was characterized using scanning electron microscopy (SEM). SEM images of the GelMA-infiltrated PCL scaffolds showed that the surface of the scaffolds was smooth and free of defects (Figure 4A). Overall, the infiltration of electrospun PCL–CPN scaffolds with GelMA resulted in a significant change in fiber diameter and morphology. The GelMA-infiltrated PCL scaffolds had a smoother surface and larger fiber diameter distribution, which may have implications for their mechanical properties and biocompatibility.

FTIR is a powerful analytical technique used to identify functional groups in chemical compounds. In this study, we observed the peaks of GelMA in the FTIR spectra of electrospun PCL–CPN scaffolds after GelMA impregnation (Figure 4B), indicating successful infiltration and incorporation of GelMA into the scaffold. The FTIR spectra of GelMA typically exhibit characteristic peaks corresponding to its functional groups. For instance, the peak at around 1650 cm^−1^ corresponds to the amide I band, which arises from the stretching vibrations of the C=O group in the peptide backbone [43,44]. The peak at around 1540 cm^−1^ is attributed to the amide II band, which arises from the bending vibrations of the N-H and C-N groups [43,44]. The peak at around 1230 cm^−1^ corresponds to the C-O stretching vibrations of the ester group [45]. Several studies have utilized FTIR spectroscopy to confirm the presence of GelMA in electrospun scaffolds. For example, Coimbra et al. employed FTIR spectroscopy to confirm the presence of GelMA in electrospun PCL/GelMA scaffolds, while Zhao et al. confirmed the presence of GelMA in electrospun PCL/GelMA scaffolds using FTIR spectroscopy [46,47]. The presence of GelMA in the FTIR spectra of electrospun PCL scaffolds containing CPN after GelMA infiltration suggests successful penetration and incorporation of GelMA into the scaffold [48,49]. The peaks of GelMA observed in the FTIR spectra indicate that the functional groups of GelMA are present in the scaffold, which can enhance its mechanical properties, biocompatibility, and cell adhesion [50].

Upon GelMA impregnation, we observed a 10–20% weight increase in the scaffold, indicating successful infiltration and incorporation of GelMA into the scaffold (Figure 4C). The weight increase observed in our study could be attributed to the impregnation of GelMA into the scaffold, which can increase the weight of the scaffold [51]. Several studies have reported successful incorporation of GelMA into electrospun scaffolds. For example, Oliveira Lobo et al. reported that GelMA-crosslinking in electrospun PCL scaffolds improved the mechanical properties and cell adhesion of the scaffolds [52]. Similarly, GelMA-infiltrated electrospun PHBV scaffolds were used for wound healing applications [53]. 

The presence of calcium, an indication of the presence of CPN, in the scaffolds was confirmed through alizarin red staining (Figure 4D,E). Alizarin red staining is a commonly used method to assess calcium deposition or mineralization [54]. The higher formation of alizarin complex observed in scaffolds loaded with 2% to 6% CPN suggests successful incorporation of CPN into the electrospun PCL scaffolds. The staining confirmed the presence of calcium in the scaffolds, which indicates the presence of calcium-containing materials (i.e., the CPN) within the scaffolds.

### 3.3. Mechanical Properties of the Composite Self-Oxygenating Scaffolds

**Figure 5:**
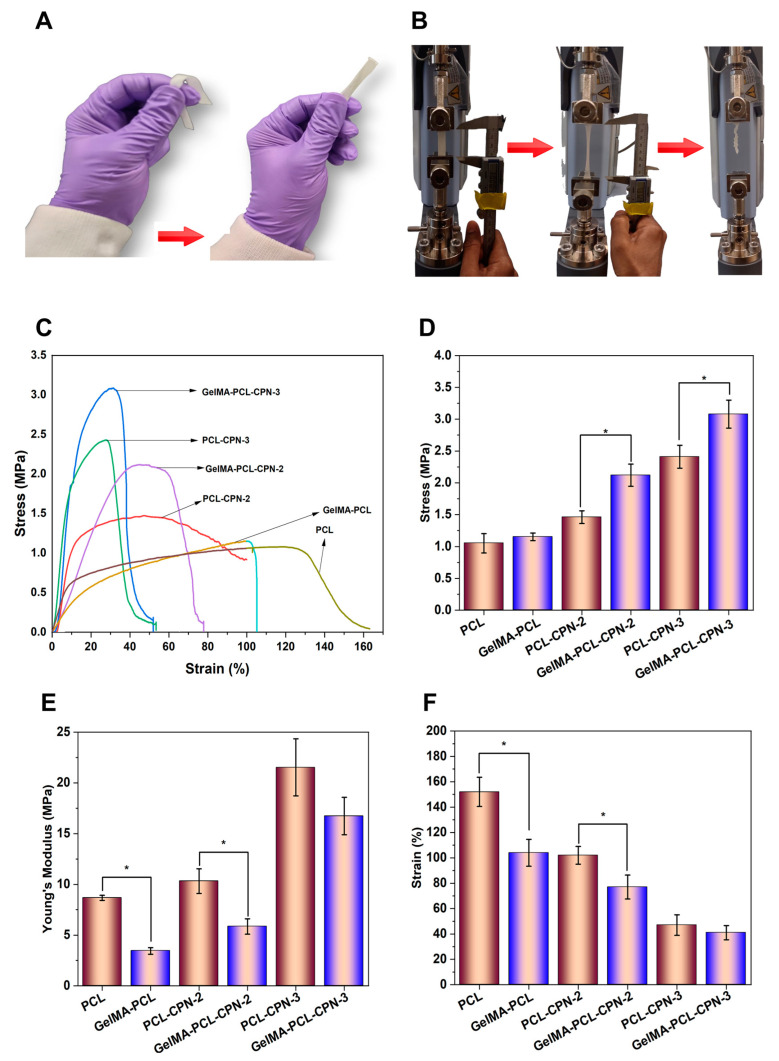
Mechanical property of the scaffolds. (**A**) Scaffold before GelMA infiltration and crosslinking (left) and after (right). (**B**) Progression of the tensile strength test. First scaffolds are loaded (left) and stretched (center) until failure occurs (right). (**C**) Stress–strain curves of the scaffolds. (**D**) Comparison of the maximum stress at failure. (**E**) Young’s modulus the scaffolds. (**F**) Comparison of the maximum strain at break. Statistically significant differences (*p* ≤ 0.05) are indicated with (*) in the figures.

Figure 5 illustrates the tensile mechanical testing results of the electrospun PCL scaffolds loaded with CPN and scaffolds subsequently infiltrated with GelMA. The addition of GelMA into the pores of the PCL–CPN matrix significantly impacted the mechanical behavior of the resulting scaffolds, leading to an improvement in their mechanical properties (Figure 5A). 

Upon tensile testing, the scaffolds displayed gradual deformation and subsequent break (Figure 5B). A stress–strain behavior characteristic of electrospun materials, with a linear elastic region at low strain and deformation before breaking, was observed (Figure 5C) [55]. However, the incorporation of CPN fillers into the PCL–GelMA matrix influenced the mechanical properties of the composite scaffolds. The tensile mechanical testing of the CPN-loaded PCL scaffolds revealed that the highest tensile strength was achieved at a 3% *w*/*w* CPN concentration (Figure 5D. The addition of CPNs above 1% *w*/*w* concentration resulted in a strong reinforcing effect, increasing the tensile modulus (Figure 5E) and inhibiting polymer drawing while decreasing elongation at break (Figure 5F). However, an increase in CPN content in the PCL beyond 3% *w*/*w* led to a reduction in tensile strength, elongation at break, and Young’s modulus. The decrease was particularly noticeable in PCL–CPN-6 scaffolds, as reported previously [29]. This finding can be a result the Payne effect, a non-linear relationship between stress and strain in reinforced polymer systems. This can be due to factors such as particle agglomeration, particle alignment, polymer-chain stretching, and filler–filler interactions under high strains or stresses [56].

It is essential to optimize both the concentration and proper dispersion of CPN fillers within the PCL matrix to achieve the desired mechanical properties for oxygen-generating scaffolds. However, the infiltration of GelMA into the pores of the CPN-loaded PCL scaffolds significantly improved the mechanical strength of the scaffolds. Thus, GelMA plays a critical role in enhancing the tensile strength and elasticity of the scaffolds, and the concentration and dispersion of CPN fillers should be carefully considered to achieve optimal results. The mechanical properties of scaffolds play an essential role in tissue engineering, and they must meet certain requirements to ensure successful regeneration.

When a material is infiltrated with another material, the infiltration layer can change the mechanical properties of the base material [52]. In the case of electrospun PCL–CPN scaffolds infiltrated with GelMA, the GelMA had a significant impact on the tensile behavior of the scaffolds, improving their mechanical properties. The base material of electrospun PCL–CPN scaffolds had varying mechanical properties depending on the concentration and dispersion of CPN fillers. The improvement in mechanical properties after GelMA infiltration could be due to the individual fibers of the electrospun scaffold being bonded together by the glue-like GelMA hydrogel, providing additional resistance against external forces, and limiting the deformation of the scaffold before breaking. Additionally, the homogeneous infiltration of the GelMA throughout the scaffold matrix could have acted as impregnation, further reducing the extent of deformation under tensile load. On the other hand, the infiltration material may have a different stiffness than the scaffold, leading to a decrease in overall stiffness of the system. This decrease in stiffness can result in a decrease in Young’s modulus. Moreover, the infiltrated material may limit the ability of the scaffold to deform before breaking, leading to a decrease in elongation at break. Therefore, the role of GelMA as an impregnation material is vital in improving the tensile strength and elasticity of the electrospun PCL–CPN scaffolds, and the concentration and dispersion of CPN fillers should be carefully considered to achieve optimal results.

The results of the tensile testing indicated that the addition of CPN led to an increase in the tensile modulus and ultimate tensile strength of the PCL scaffolds infiltrated with GelMA. The improved mechanical properties could be attributed to the homogeneous distribution of the CPN throughout the scaffold matrix, which acted as impregnation and reduced the extent of deformation under tensile load. Wutticharoenmongkol et al. have reported similar findings, where the incorporation of nanoparticles in PCL scaffolds resulted in significantly enhanced mechanical properties, compared to PCL scaffolds without nanoparticles [57]. The authors attributed the improvement to the homogeneous dispersion of the nanoparticles, as evidenced by the increasing mechanical strength observed as the nanoparticle concentration increased. Additionally, when implanted in the animal body, the implantation site experiences various mechanical stresses caused by movement, tissue growth, or physiological forces. Polymers with higher tensile strength can effectively withstand these mechanical stresses, thereby maintaining their structural integrity for a longer period. This resistance to mechanical stress can slow down the degradation process and proves beneficial for applications such as bone-tissue engineering, where gradual degradation of scaffolds is desirable [58,59].

### 3.4. Enhanced Swelling Behavior of Electrospun PCL Scaffolds by GelMA Impregnation

**Figure 6:**
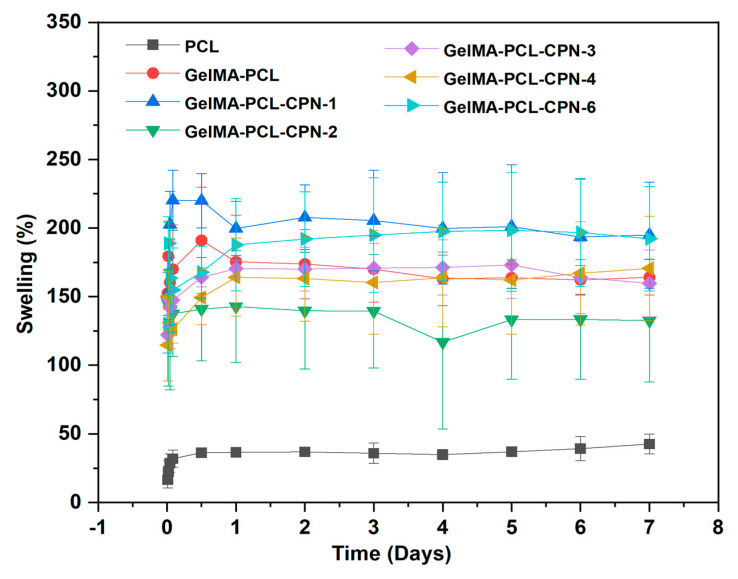
Swelling behavior comparison of GelMA–PCL and GelMA–PCL–CPN scaffolds in PBS.

Electrospun PCL scaffolds are widely used in biomedical applications due to their high porosity, biocompatibility, and mechanical properties. However, the hydrophobic nature of PCL limits its use in tissue engineering applications, as it allows for limited cell adhesion and proliferation. To overcome this limitation, GelMA was impregnated into the PCL scaffolds, as it has been reported to enhance the hydrophilicity as well as swelling of the scaffolds and support cell attachment and proliferation. We investigated the effect of GelMA impregnation on the swelling behavior of electrospun PCL scaffolds. The swelling behavior of the scaffolds was measured at different time points to determine the effect of GelMA infiltration on the hydrophilicity as well as weight gain of the scaffolds. The results of this study showed that the swelling of the electrospun PCL scaffolds significantly increased over time as the GelMA was infiltrated (Figure 6). The increase in swelling can be attributed to the hydrophilic nature of GelMA, which increases the water uptake of the scaffold. This increased swelling is beneficial for tissue engineering applications, as it enhances the transport of nutrients and waste products within the scaffold and supports cell proliferation. 

Overall, the results of swelling study suggest that the GelMA-infiltrated PCL scaffolds have improved hydrophilicity and swelling behavior, which make them suitable for tissue engineering applications. 

### 3.5. Oxygen Generation Capacity of the Self-Oxygenating Scaffolds

The results of the study indicate that the immediate release of CPN from PCL scaffolds was prevented by GelMA infiltration, thereby enabling sustained oxygen release from the scaffolds. The sustained release of oxygen is highly advantageous for bone-tissue engineering, as it provides a continuous supply of oxygen to support cell proliferation and differentiation [60].

**Figure 7:**
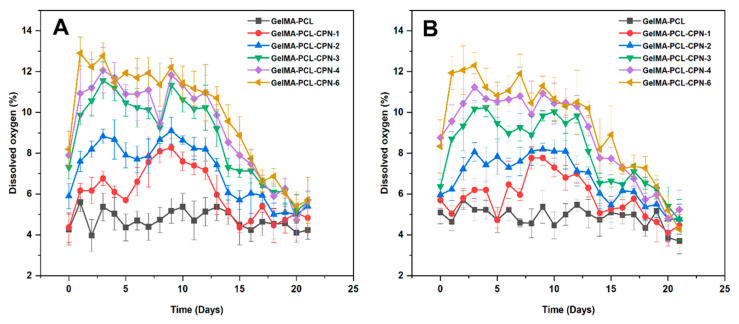
Release of oxygen (O_2_) from the GelMA–PCL–CPN scaffolds under hypoxia for 21 days. (**A**) Graph showing the release of oxygen from the cell-free scaffolds. (**B**) Oxygen release from the cell-seeded scaffolds.

A recent investigation demonstrated that GelMA infiltration can provide a more sustained release of oxygen from scaffolds, which could be highly beneficial for bone regeneration [61]. In this regard, we conducted experiments to assess the oxygen release capacity of the scaffolds by placing 25 mg of scaffolds in Dulbecco’s modified Eagle’s medium (DMEM) for 21 days under hypoxic conditions with oxygen levels kept at 4–6%. The experiment involved testing the oxygen-release capacity of pre-osteoblast-seeded scaffolds and those without cells, both under hypoxia (Figure 7). The oxygen release was lower for cell-seeded scaffolds due to the oxygen consumption by the cells, as expected. The PCL–GelMA scaffolds loaded with 1% and 2% CPN showed similar oxygen release with and without cell seeding, as the cell proliferation was minimal. Similarly, when loaded with 6% CPN, cell proliferation was low due to the cytotoxic response to CPN at higher concentrations.

These results suggested that the burst release of oxygen from PCL scaffolds was prevented by GelMA impregnation, enabling sustained oxygen release. Sustained release of oxygen is advantageous for bone-tissue engineering as it augments cell proliferation and differentiation [62]. The experiment involved placing 25 mg of scaffolds in DMEM for 21 days under hypoxic conditions with oxygen levels at 4–6%. The results showed that even 1% *w*/*w* CPN-loaded scaffolds exhibited significantly higher oxygen levels (7–15% oxygen) under hypoxia compared to the neat PCL scaffolds (4–7.5% oxygen). PCL scaffolds loaded with 3–6% *w*/*w* CPN exhibited an initial high oxygen release that peaked on day four (up to 18% oxygen), but the oxygen level dropped to 10–12% after seven days.

The high surface area of CPN promotes the release of H_2_O_2_, which is further converted into oxygen by catalase in the culture media. The presence of loosely bound nanoparticles near the scaffold’s surface is responsible for the higher initial release of oxygen in 4 and 6% *w*/*w* CPN-loaded scaffolds. This finding is consistent with other studies indicating that a higher initial release of oxygen is due to the presence of loosely bound nanoparticles near the scaffold’s surface. A sustained release of oxygen follows the initial release, indicating that the scaffolds can maintain oxygen release over an extended period. The sustained release of oxygen indicates that the scaffolds can maintain oxygen release over a prolonged period, facilitating a continuous supply of oxygen to the site of injury. Oxygen is crucial for cellular metabolism and proliferation, and a sustained release of oxygen from scaffolds can expedite the recovery process and promote bone regeneration [63].

### 3.6. The Composite Self-Oxygenating Scaffolds Improved Cell Adhesion, Viability, and Proliferation under Hypoxia

Hypoxia, or a lack of oxygen supply, can have detrimental effects on cell survival and proliferation, particularly in the context of tissue engineering. In bone-tissue engineering, the use of oxygen-generating scaffolds can provide a solution to the issue of hypoxic conditions in the defect site, promoting bone regeneration [25]. Oxygen-generating scaffolds release oxygen into the surrounding environment, which can enhance cell survival and proliferation, particularly under hypoxic conditions [64]. In bone-tissue engineering, the use of oxygen-generating scaffolds is particularly important, as bone cells require a constant supply of oxygen to maintain their function and promote bone regeneration [65]. The sustained release of oxygen from these scaffolds can improve the survival and proliferation of bone cells, leading to enhanced bone formation. 

**Figure 8:**
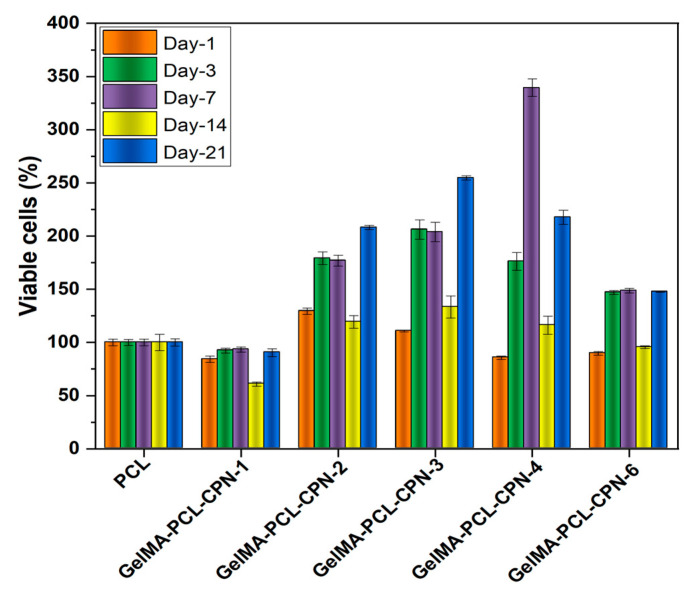
Proliferation of the pre-osteoblast cells on GelMA-PCL–CPN scaffolds under hypoxia. Results of the MTT assay on the self-oxygenating scaffolds cultured with pre-osteoblasts for 21 days.

**Figure 9:**
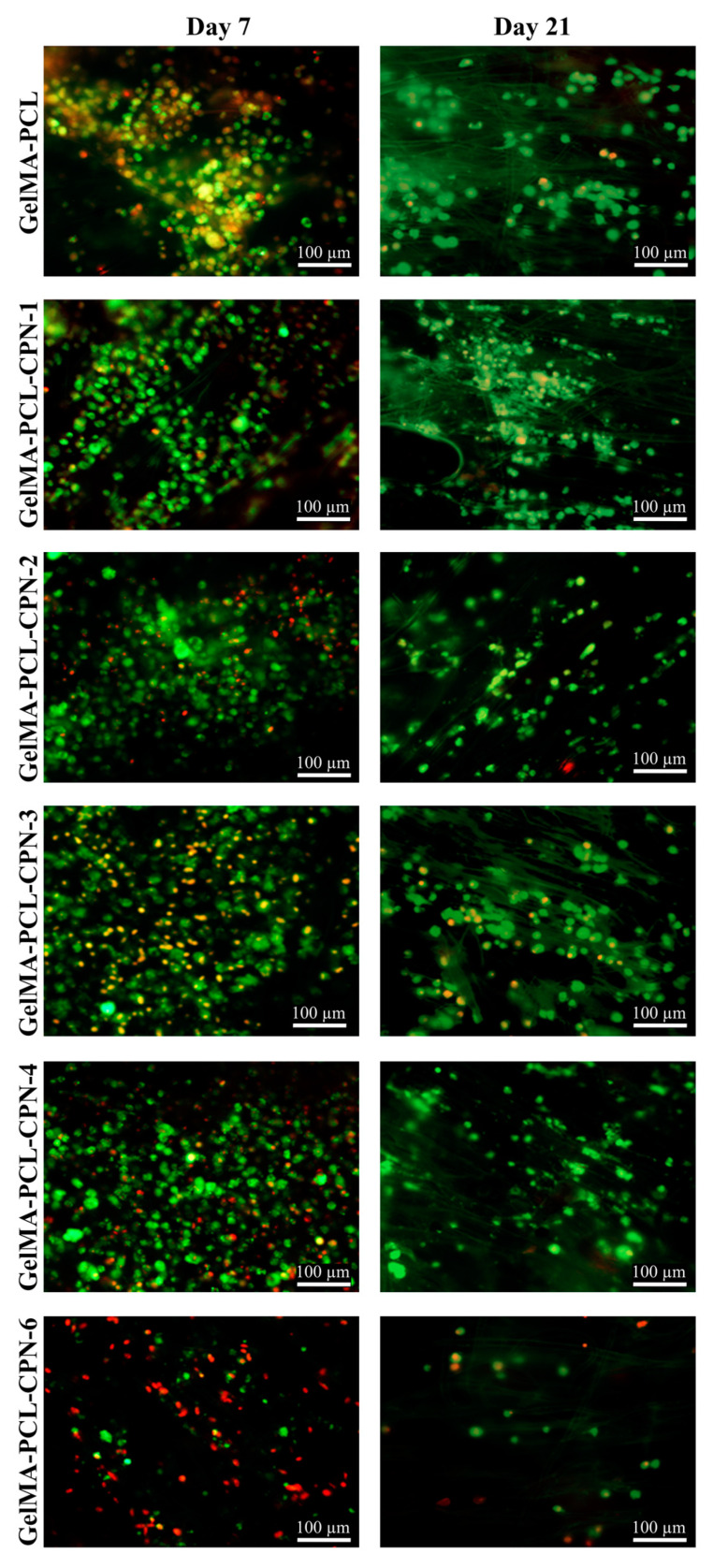
Viability of the pre-osteoblast cells on GelMA-PCL–CPN scaffolds under hypoxia. Live/Dead assay showing the live (green) and dead (red) pre-osteoblast cells proliferating on the scaffolds.

In order test our hypothesis, we cultured pre-osteoblast cells on GelMA–PCL as well as GelMA-PCL–CPN scaffolds under hypoxic conditions for 21 days. MTT cell viability assay showed that the composite GelMA-PCL–CPN scaffolds provided higher amount of cell proliferation under hypoxic conditions (Figure 8). In addition, the number of viable cells which attached and proliferated on the GelMA-PCL–CPN scaffolds were greater than that of the blank GelMA–PCL scaffolds (Figure 9). Specifically, 2 to 4% *w*/*w* CPN loading in the scaffolds resulted in the proliferation of large number of pre-osteoblast up to 21 days. However, a higher loading of CPN in the scaffolds resulted in a decrease in cell proliferation and viability, as demonstrated in the case of GelMA-PCL–CPN-6. This can be attributed to the potential adverse effects of CPN at higher concentrations, as reported in various studies [7]. Specifically, a significant number of dead cells were observed on day 7 for GelMA–PCL–CPN-6. Additionally, the overall cell density on GelMA–PCL–CPN-6 was considerably lower at 21 days of the study. This decrease in cell density on day 21could be attributed to the detachment and loss of dead cells from the scaffolds during the extended culturing period and the necessary media changes.

Previous studies have demonstrated that oxygen delivery is a key factor in promoting bone-tissue regeneration. For instance, Peng et al. showed that the sustained release of oxygen from magnesium-peroxide-containing scaffolds improved osteogenic differentiation of bone marrow-derived mesenchymal stem cells (BMSCs) in vitro and increased bone formation in a rat femoral condyle defect model [66]. Similarly, Touri et al. reported that the use of oxygen-generating scaffolds enhanced bone-tissue regeneration in a rabbit radius defect model [60]. They used robocasted biphasic calcium phosphate (BCP) containing CaO_2_ to deliver oxygen to the defect site [60]. These studies are consistent with our findings and demonstrate the potential of oxygen-generating scaffolds for bone-tissue engineering applications. Thus, oxygen-generating scaffolds have promising potential in bone-tissue engineering applications due to their ability to protect cells from hypoxic cell death and promote cell proliferation under hypoxic conditions. The use of these scaffolds can improve the success of bone regeneration, providing a promising solution for the treatment of bone defects. The GelMA was impregnated into the PCL–CPN scaffold as it enhances the cytocompatibility of the synthetic polymeric scaffolds [31]. GelMA is derived from gelatin, a natural protein found in various connective tissues, and can be crosslinked to form a hydrogel. Accordingly, our study showed that the addition of GelMA to the PCL–CPN scaffold supported cell adhesion and proliferation, promoting cell migration. Another study by Buyuksungur et al. (2021) employed dental pulp stem cell (DPSC)-laden GelMA printed alongside PCL, which was shown to enhance the osteogenic differentiation of DPSCs [67]. 

Overall, the findings of this study indicate that self-oxygenating electrospun PCL–CPN scaffolds, combined with GelMA, hold promise for applications in bone-tissue engineering. However, additional studies are required to investigate the expression of genes related to bone formation, as well as in vivo implantation studies. In terms of physico-mechanical properties and the proliferation of pre-osteoblasts under hypoxic conditions, the GelMA-impregnated scaffold loaded with 3% *w*/*w* CPN shows potential for further detailed investigation.

## 4. Conclusions

In conclusion, the study demonstrated the potential of electrospun PCL scaffolds containing calcium peroxide nanoparticles (CPN) impregnated with GelMA as a promising biomaterial for tissue engineering applications. The incorporation of CPN in the scaffolds allowed for the sustained release of oxygen, improving cell viability, proliferation, and osteogenic differentiation. The improved mechanical properties of the scaffolds and their ability to promote cell proliferation under hypoxia suggest their potential in facilitating tissue regeneration. The study findings are consistent with existing literature on the benefits of oxygen delivery in promoting bone-tissue regeneration and the use of PCL and GelMA in scaffold fabrication. The addition of CPN further enhanced the mechanical strength and stiffness of the scaffold without causing adverse effects on cell proliferation at optimal concentrations. The study results hold significant implications for future research in bone-tissue engineering, particularly for the development of biocompatible and efficient biomaterials that can enhance tissue regeneration.

## Data Availability

The data used to support the findings are included within the article.
